# EXPLORING THE ASSOCIATION OF SLEEP WITH COGNITIVE DOMAINS AMONG PEOPLE LIVING WITH DEMENTIA

**DOI:** 10.1093/geroni/igad104.3332

**Published:** 2023-12-21

**Authors:** Stevie Lewis, Charmi Patel, Deborah Jehu

**Affiliations:** Augusta University, Augusta, Georgia, United States; Augusta University, Augusta, Georgia, United States; Augusta University, Augusta, Georgia, United States

## Abstract

People living with dementia (PWD) commonly experience sleep disruptions, which impacts mental alertness, increases daytime sleepiness, and results in poorer quality of life. While sleep preserves cognitive function among PWD, the relationship between sleep and different cognitive domains is unclear. Our objective was to explore the association between sleep and cognition domains among PWD. Twenty-three PWD completed a battery of cognitive tests (global cognition: Montreal Cognitive Assessment; executive function: Trail-Making Test (TMT), Digit Span Forward Test; perception and orientation: Benton Judgement of Line Orientation Test (BJLO); language: Boston Naming Test; learning and memory: Rey Auditory Verbal Learning Test; complex attention: Digit Symbol Substitution Test) at two residential care facilities. Participants wore a motion watch on their wrist for 7 days to monitor sleep. Participants were primarily male (74%), white (87%), and 48% had unspecified dementia. The average number of hours of sleep per night was 4.6±1.2 hours. The overall regression model for sleep was significant (R2=0.80, F(10,12)=4.68, RMSE=42.32, p< 0.05). More sleep was associated with better perception and orientation (BJLO:β=0.57, p=0.03), greater sleep efficiency (β=0.93, p=0.01), longer wake after sleep onset (β=1.19, p 0.002), and there was a trend for better executive function (TMT:β=-0.34, p=0.09). PWD have inadequate sleep. Greater amounts of sleep seem to preserve perception and orientation and may play a protective role for executive function, but more evidence is needed. Regularly assessing sleep may be useful in screening and monitoring cognitive changes among PWD in residential care facilities.

